# Dehydrocostus lactone inhibits *Candida albicans* growth and biofilm formation

**DOI:** 10.1186/s13568-023-01587-y

**Published:** 2023-08-04

**Authors:** Jingxiao Zhang, Jian Sun, Yu Zhang, Min Zhang, Xin Liu, Longfei Yang, Yongjie Yin

**Affiliations:** 1 Department of Emergency and Critical Care Medicine, The Second Hospital of Jilin University, 218# Ziqiang Street, Changchun, 130041 China; 2 Department of Clinical Laboratory, The Second Hospital of Jilin University, 218# Ziqiang Street, Changchun, 130041 China; 3 Eye Center, The Second Hospital of Jilin University, 218# Ziqiang Street, Changchun, 130041 China; 4 Jilin provincial key laboratory on molecular and chemical genetic, The Second Hospital of Jilin University, 265# Ziqiang Street, Changchun, 130041 China

**Keywords:** *Candida albicans*, Dehydrocostus lactone, Antifungal, Biofilm, Reactive oxygen species (ROS)

## Abstract

*Candida albicans* infections are threatening public health but there are only several antifungal drugs available. This study was to assess the effects of dehydrocostus lactone (DL) on the *Candida albicans* growth and biofilms Microdilution assays revealed that DL inhibits a panel of standard *Candida* species, including *C. albicans*, as well as 9 *C. albicans* clinical isolates. The morphological transition of *C. albicans* in RPMI-1640 medium and the adhesion to polystyrene surfaces can also be decreased by DL treatment, as evidenced by microscopic, metabolic activity and colony forming unit (CFU) counting assays. The XTT assay and microscopy inspection demonstrated that DL can inhibit the biofilms of *C. albicans*. Confocal microscopy following propidium iodide (PI) staining and DCFH-DA staining after DL treatment revealed that DL can increase the membrane permeability and intracellular reactive oxygen species (ROS) production. *N*-acetyl-cysteine could mitigate the inhibitory effects of DL on growth, morphological transition and biofilm formation, further confirming that ROS production induced by DL contributes to its antifungal and antibiofilm effects. This study showed that DL demonstrated antifungal and antibiofilm activity against *C. albicans*. The antifungal mechanisms may involve membrane damage and ROS overproduction. This study shows the potential of DL to fight *Candida* infections.

## Key points

Dehydrocostus lactone inhibits the adhesion, morphological transition and biofilms of *Candida albicans.*

Dehydrocostus lactone causes *C. albicans* cell membrane damages.

Dehydrocostus lactone induces ROS overproduction in *C. albicans* cells and NAC supplementation can attenuate the activity of dehydrocostus lactone against *C. albicans*.

## Introduction

In past decades, fungal infections caused by *Candida* species increase progressively and are the major cause of fungemia involving central venous catheter (Bandara et al. 2017; Liu et al. 2017). Among *Candida* infections, although cases by non-albicans *Candida* species are increasing, *C. albicans* infections take the large part, which was supported by a recently-published study that surveys 4010 isolates from candidemia patients of 77 hospital over three years in China (Xiao et al. 2020). The polymorphic *C. albicans*, which is able to grow as a yeast, pseudohypha or hypha, can colonize on skin, oral cavity, vagina and gastrointestinal tract without causing diseases. Once the immunity was compromised by drugs or other factors, infections, such as oral thrush, vulvovaginal candidiasis and candidemia, may be found in these sites and bloodstreams (Bandara et al. 2017). The available antifungal drugs are only limited to azoles, echinocandins and polyenes (Robbins et al. 2017). The prevalence of antifungal resistance is also increasing, while the multidrug-resistant *Candida* species is emerging (Robbins et al. 2017; Xiao et al. 2020). This situation, together with the dearth of antifungal drugs (Perfect 2017), makes it imperative to develop novel antifungal therapies.

Natural products have been increasingly popular in some medical research fields, such as antitumor (Di et al. 2014; Maryam et al. 2017) and antimicrobial studies (Moloney 2016; Liu et al. 2017; Yang et al. 2018b, 2022a), partly because the traditional medicine were generally thought to be less toxic and to harbor a huge pool of diversified compounds. Dehydrocostus lactone (DL) is a bioactive sesquiterpene lactone that can be isolated from the herb *Aucklandia lappa* Decne (Yuan et al. 2022). Besides its antitumor activity (Jiang et al. 2015; Zhang et al. 2020) and anti-inflammatory activity (Pyun et al. 2018; Wu et al. 2021; Yuan et al. 2022), DL also shows antibacterial (including antimycobacterial activity) (Cantrell et al. 1998; Ibrahim et al. 2019; Deyno et al. 2020) and larvicidal activities (Neves et al. 1999). In addition, DL has exhibited antifungal effects on some human and plant pathogenic fungi, including *Cladosporium Cucumerinum* (Neves et al. 1999), *Fusarium graminearum* (Mousa et al. 2016), *Cunninghamella echinulate* (Barrero et al. 2000), *Botrytis cinerea* (Wedge et al. 2000), *Colletotrichum acutatum* (Wedge et al. 2000), *Colletotrichum fragariae* (Wedge et al. 2000), *Colletotrichum gloeosporioides* (Wedge et al. 2000), *Aspergillus niger* (Shin and Kim, 2016), *Aspergillus clavatus* (Shin and Kim, 2016), *Candida parapsilosis* (Shin and Kim, 2016), *Rhizopus oryzae* (Shin and Kim, 2016), *Saccharomyces cerevisiae* (Shin and Kim, 2016), *C. albicans* (Shin and Kim, 2016), *Candida tropicalis* (Shin and Kim, 2016), *Candida glabrata* (Shin and Kim, 2016), *Cryptococcus neoformans* (Shin and Kim, 2016), *Pichia guilliermondii* (Shin and Kim, 2016) and *Ascosphaera apis* (Shin and Kim, 2016).

Although DL has been shown to inhibit the fungus *C. albicans* as assayed by bioautography, microdilution and agar diffusion method (Neves et al. 1999; Shin and Kim, 2016), there remains to be explored about its antifungal mechanisms and its effects on *C. albicans* virulence factors. In this research we exploreed the antifungal activity of DL on *C. albicans* and its virulence traits (morphological transition, adhesion and biofilm formation), as well as the preliminary mechanisms underlying its antifungal effects.

## Materials and methods

### Strains and cultures

Six standard *Candida* strains and *C. albicans* clinical isolate 1–9 (from the clinical laboratory of our hospital) (listed in Table 1) were used to assess the activity of DL. All these strains were kept on YPD (abbreviation for the medium containing 1% yeast extract, 2% peptone and 2% dextrose) agar at 4 °C and sub-cultured in YPD medium for about 18 h (28 °C and 140 rpm) for proliferation. The temperature for all the assays was 37 °C.

**Table 1:** Antifungal susceptibility of DL and AmB against *Candida* species

Strains	DL		AmB	
	MIC (µg/mL)	MFC (µg/mL)	MIC (µg/mL)	MFC (µg/mL)
*C. albicans* SC5314	64	128	1	2
*C. albicans* ATCC 10231	64	256	1	2
* C. albicans isolate 1*	64	> 256	1	2
* C. albicans isolate 2*	64	> 256	1	2
* C. albicans isolate 3*	64	256	1	2
* C. albicans isolate 4*	64	128	1	2
* C. albicans isolate 5*	64	> 256	1	2
* C. albicans isolate 6*	64	128	1	2
* C. albicans isolate 7*	64	> 256	1	2
* C. albicans isolate 8*	64	128	1	2
* C. albicans isolate 9*	64	128	1	2
* C. parapsilosis* ATCC 22019	64	128	1	2
* C. tropicalis* ATCC 7349	64	128	1	2
* C. krusei* ATCC 6258	32	64	1	2
* C. glabrata* ATCC 2001	32	64	1	2

Dehydrocostus lactone (SD8110), *N*-acetyl-cysteine (NAC) and ROS detection kit (CA1410) was bought from Solarbio Biotech (Beijing, China). Methyl-tretrazolium salt (MTT), and amphotericin B (AmB) were from Shanghai Sangon. Propidium iodide (PI), 2, 3-bis (2-methoxy-4-nitro-5-sulfophenyl)-2 H-tetrazolium-5-carboxanilide (XTT) and Calcofluor White (CFW) were Sigma-Aldrich (Shanghai, China) products.

### Antifungal susceptibility assay

In this assay, the CLSI-M27-A3 recommended microdilution method in 96-well plates was used as we described previously (Yang et al. 2018c), to determine the minimal inhibitory concentration (MIC) and minimal fungicidal concentration (MFC). 100 µL fungal cells (2 × 10^3^ cells/mL) in 96-well plate were treated with DL (0-256 µg/mL) for 24 h at 37 °C. The lowest concentration at which no visual growth in wells were defined as MIC. Cultures in wells containing higher DL than or equal to MIC were plated on YPD agar to allow for growth at 37 °C for 24 h. The lowest concentration at which no colony were grown on agar were set as MFC.

### Morphological transition

In this assay, a density of 10^6^ cells/mL (*C. albicans* SC5314) in RPMI-1640 medium was used to induce the transition from yeasts to hyphae. Treatment with 0, 16, 32 and 64 µg/mL DL for 4 h at 37 °C preceded the photographing of *C. albicans* morphologies with an inverted microscope (Olympus IX81, Japan) at 40×.

### Adhesin to polystyrene surfaces

*C. albicans* cells in RPMI-1640 medium were treated with various concentrations of DL for 90 min at 37 °C, followed by washing with sterile PBS for three times. Then MTT (5 mg/mL in sterile PBS) tests were performed to evaluate the relative adhesion as follow: adding 10 µL MTT into each well followed by 4 h incubation at 37 °C, emptying wells, adding 100µL DMSO and determining OD_570nm_ of each well (Han et al. 2020).

To exclude the possibility that the reduced adhesion was due to the reduced viable cells that caused by DL treatment, the viable cells after 90-minute treatment of DL were calculated by serial dilution and plating on Sabouroud dextrose (SD) agar. Additionally, the adherent cells left on polystyrene plates were subjected to CFW staining (20 µg/mL), followed by microscopic record (Olympus IX81, Japan) at 40× (Yang et al. 2022a).

### Biofilm formation and development

*C. albicans* SC5314 biofilms were grown in micro-well plates statically as previously described (Yang et al. 2022b). Fungal cells propagating for 18 h at 28 °C, 140 rpm were collected by centrifugation and diluted with RPMI-1640 medium to 10^6^ cells/mL. 100 µL such suspension was added into each well of 96-well plates which were kept at 37 °C statically for 24 h to allow biofilm formation. Adding DL in each well immediately after seeding *C. albicans* suspension was performed to evaluate the influence of DL on biofilm formation. 24 h later, XTT reduction assay was performed to determine the metabolic activity of biofilms formed in the presence of DL. In addition, 24-hour old biofilms formed in the absence of drugs were challenged by DL-containing fresh medium for twenty-four hour, followed by XTT tests to evaluate the activity against preformed biofilms. XTT assay conditions: biofilms in each well were washed with PBS and then exposed to 100 µL sterile XTT; 2 h later, 75 µL supernatant of each well were transferred to a new plate and determined at OD_490nm_ (Yang et al. 2022b).

### CFW staining of biofilms

To observe the influence of DL on the 3D structures of *C. albicans* biofilms, CFW staining (20 µg/mL, 10 min in dark at 37 °C) was used. After washing, the biofilms were inspected by confocal laser scanning microscope (CLSM, Olympus FV1000) at 40×. The *xyz* mode was used to scan the biofilms at a step-size of 2 μm while the slice numbers varied in accordance with the biofilm heights. Scanned data were reconstructed by Imaris 7 as we described previously (Yang et al. 2018c).

### Exopolysaccharide (EPS) determination

The determination of EPS of the preformed biofilms in 24-well plates was performed following the method we described previously elsewhere (Yang et al. 2022b), the main conditions were 200 µL 0.9% NaCl, 200 µL 5% phenol and 2 mL 0.2% hydrazine sulfate in each well containing biofilms. After incubation at 37 °C for 1 h, OD_490nm_ of each well were recorded.

### Extracellular phospholipase assay

One microliter of cell suspension was added on egg emulsion agar mixed with DL and grown for 4 days at 37 °C. A higher ratio (Pz) of the colony diameter to that of colony and precipitation zone would appear in the cells with low production of extracellular phospholipase (Yang et al. 2018c).

### PI staining

PI (a cell membrane impermeable fluorescent dye) was used at a final concentration of 10 µM to stain *C. albicans* cells (10^6^ cells/mL in RPMI-1640 medium) that were treated with DL for 4 h. The fungal cells with damaged plasma membrane would be stained by PI (10 min at 37 °C) and emit red fluorescence, which could be detected by CLSM (Olympus FV1000, at 40×) (Yang et al. 2018a).

### ROS detection

*C. albicans* cells were treated with DL for 4 h and then stained with10 µM 2’,7’-dichlorofluorescein diacetate (DCFH-DA) for half an hour in dark. After washing with PBS, fungal cells were sent for CLSM (Olympus FV1000) observation at 40×(Yang et al. 2022b).

### NAC rescue experiments

To test the contribution of ROS to the antifungal effects of DL, supplement of the antioxidant NAC (150 µg/mL) was performed in the tests of MIC, morphological transition and biofilm formation. These assays were performed under the same conditions as we mentioned above in Part of MIC assay, morphological transition assay and biofilm formation assay (Yang et al. 2018a).

### Statistical analysis

The data presented were mean ± standard derivation from triplicates in three independent assays and Student *t* test (by GraphPad Prism 6.02) were employed to analyze the statistical significance.

## Results

### Antifungal susceptibility

DL showed obvious inhibitory activities in five *Candida* species tested (Table 1). The MICs of DL were 64 µg/mL against all strains except *C. glabrata* ATCC 2001 and *C. krusei* ATCC 6258, the MIC against which were 32 µg/mL. The MFC of DL varied from 64 µg/mL (*C. glabrata* and *C. krusei*) to above 256 µg/mL (four *C. albicans* clinical isolates). Since *C. albicans* accounts for a bigger part of *Candida* infections (Tucey et al. 2018) and *C. albicans* SC5314 was the most used strain for *C. albicans* biofilm research (Yang et al. 2018b; Fathi-Hafshejani et al. 2023), we selected this strain for further studies.

### Adhesion assay

**Fig. 1:**
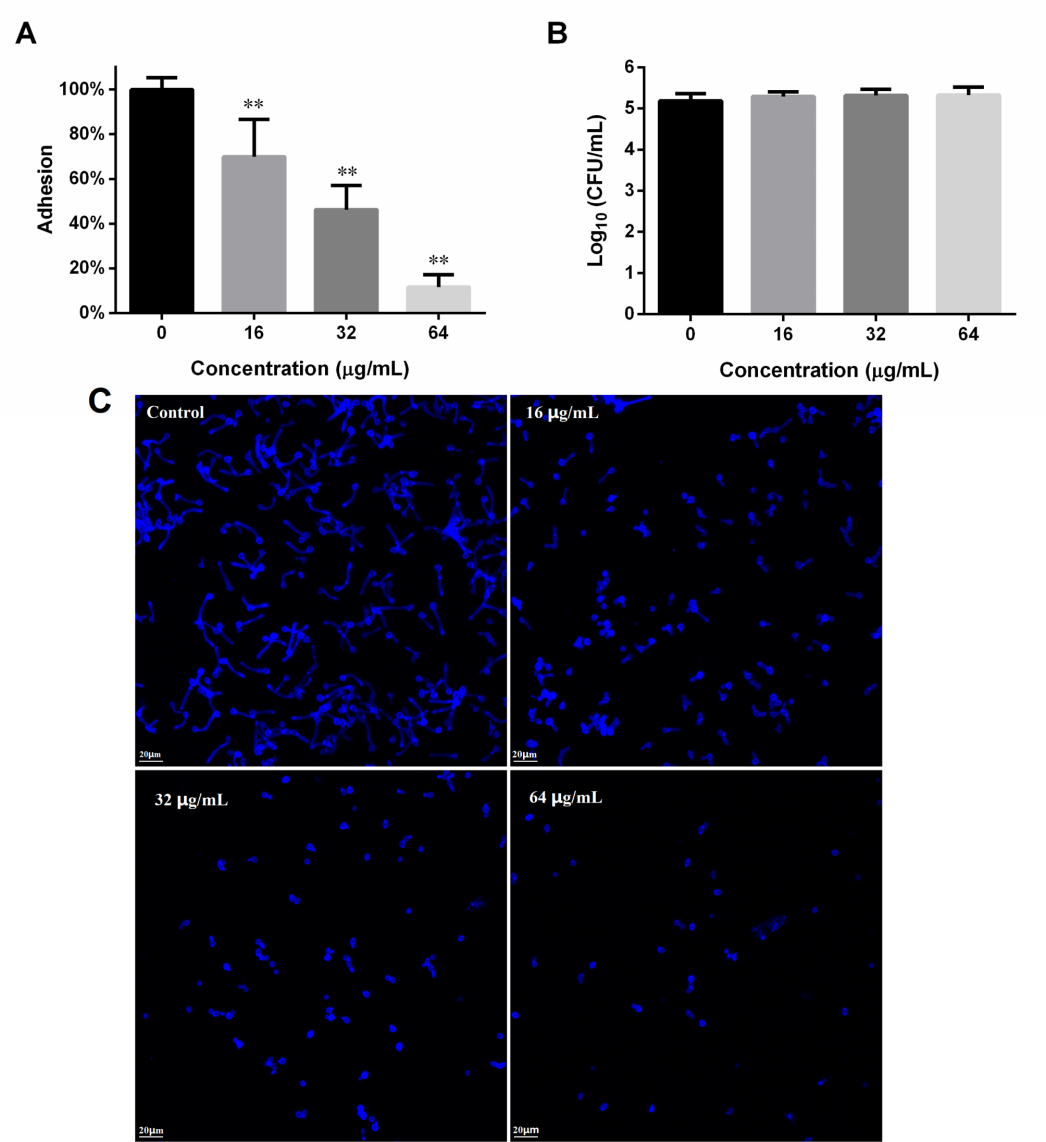
DL suppressed the adhesion of *C. albicans* to polystyrene surfaces. (**A**) After a 90-minute incubation with different concentrations of DL at 37 °C and washing with PBS, the viability of *C. albicans* cells left on the surfaces were determined by MTT. (**B**) After treating with DL for 90 min, the viable cells in the wells were evaluated by counting CFUs on SD agar after serial dilution, plating and incubation at 37 °C for 24 h. (**C**) *C. albicans* cells treated with different concentrations of DL for 90 min were washed with PBS, stained with CFW and then photographed. **, *p* < 0.01

DL inhibited *C. albicans* SC5314 adhesion to polystyrene materials (Fig. 1A). At sub-MIC 16 and 32 µg/mL, the adhesion could be decreased to 69% and 46% of the drug-free controls. At 64 µg/mL, DL can decrease the adhesion to 11% of controls. The CFU counting assay after 90-minute treatment of DL showed that DL at 16–64 µg/mL did not significantly reduce the viable cells (Fig. 1B), indicating that DL inhibits the adhesion not through reducing viable cells. To further confirm the suppression of DL on *C. albicans* adhesion, we used CFW to stain the adherent cells that have undergone DL treatment and PBS washing. As revealed by Fig. 1C, as the concentrations increased, the fungal cells left on surfaces gradually reduced.

### DL inhibited ***C. albicans*** biofilms

**Fig. 2:**
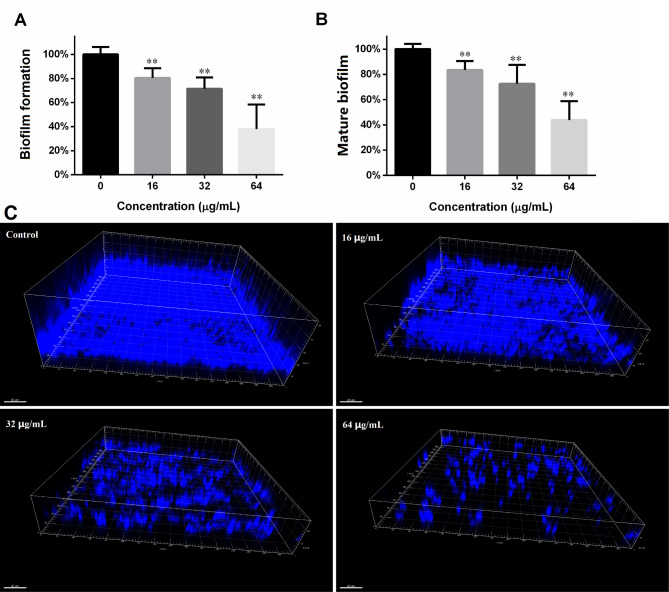
DL suppresses the formation and development of *C. albicans* biofilm. **A**: *C. albicans* biofilms formed in the presence of DL were subjected to XTT reduction assay. **B**: 24-hour old biofilms were treated with different concentrations of DL for another 24 h, followed by XTT assay. **, *p* < 0.01. **C**: *C. albicans* biofilms formed in the absence or presence of DL (16, 32 and 64 µg/mL) were stained with CFW and washed with PBS, then subjected to CLSM for the *xyz*-mode recording. The three-dimensional pictures shown were built using Imaris 7.2.3 (BitPlane, Switzerland)

As shown in Fig. 2A, treatment with 16, 32 and 64 µg/mL DL gradually decreased the metabolic activity of *C. albicans* biofilms, indicating that DL compromised biofilm formation. The similar trend and extent of metabolic activity reduce could also be seen in Fig. 2B, which suggests that DL compromised the development of *C. albicans* biofilms. In addition, the dose-dependent inhibitory effects of DL on biofilm formation was seen with microscope as well. Figure 2 C showed that the increasing DL concentration caused thinner biofilms and more space between cell clusters.

### DL inhibited the morphological transition of ***C. albicans***

**Fig. 3:**
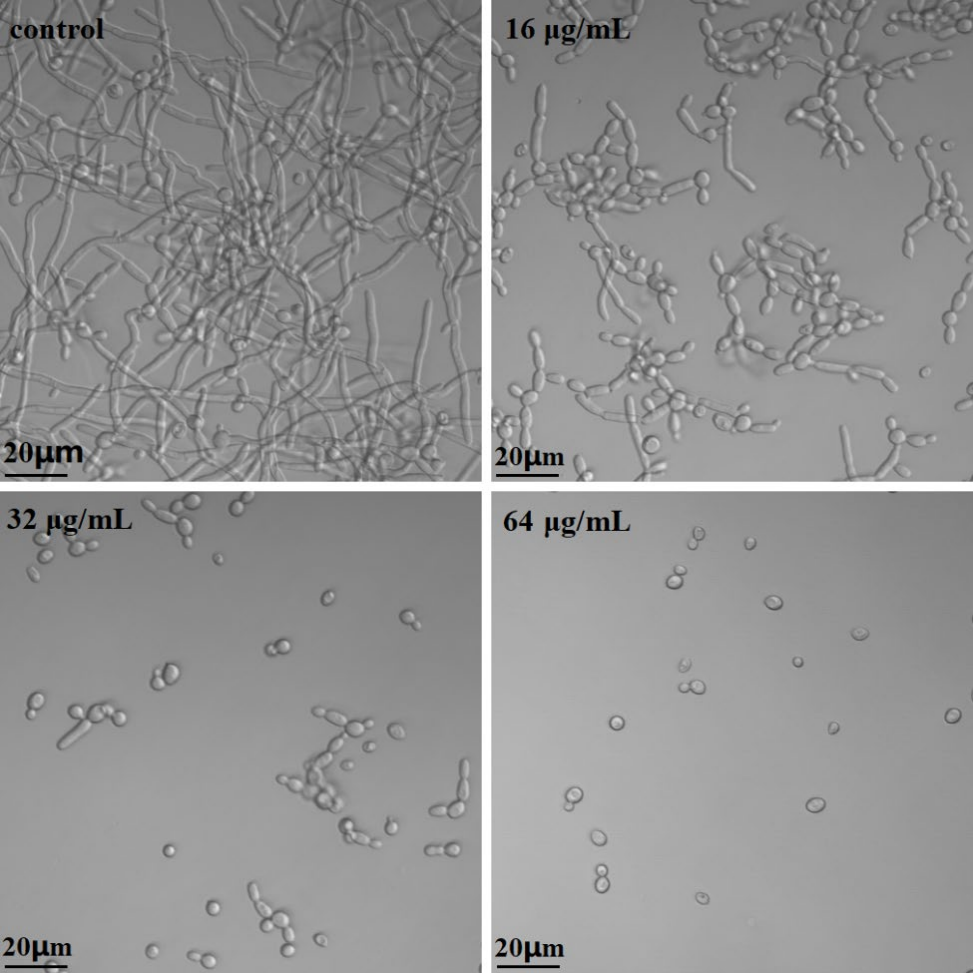
DL inhibits the yeast to hypha transition of *C. albicans* SC5314. Fungal cells (10^6^ cells/mL in RMPI-1640 medium) were exposed to 0, 16, 32 and 64 µg/mL of DL at 37 °C for 4 h and then the cell morphologies were photographed using an inverted microscope

As shown in Fig. 3, treatment with DL of 16, 32 and 64 µg/mL could obviously suppress the hyphal formation of *C. albicans* induced by RPMI-1640 medium plus 37 °C. The hyphal proportion and length decreased as DL concentrations rises. Since *C. albicans* hyphae play critical roles in tissue invasion and intracellular survival, the hyphal inhibition of DL indicates its downregulating effects on *C. albicans* infectivity.

### DL inhibited EPS and extracellular phospholipase production

**Fig. 4:**
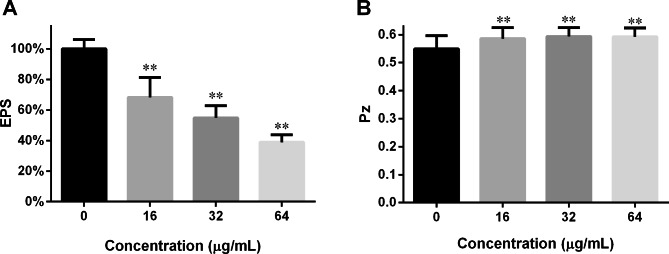
DL inhibits the production of EPS and extracellular phospholipase. **A**: Preformed *C. albicans* SC5314 biofilms were treated with 0, 16, 32 and 64 µg/mL of DL for 24 h before the determination of EPS production in biofilms through phenol-hydrazine sulfate method. **B**: Fungal cells were added on egg yolk emulsion agars supplemented with 0, 16, 32 and 64 µg/mL of DL. After 4-day incubation at 37 °C, the diameter of colony (*d*_*1*_) and that of colony plus precipitation zone (*d*_*2*_) were measured. Pz means *d*_*1*_/*d*_*2*_, while higher Pz indicates lower phospholipase production. **, *p* < 0.01

As shown in Fig. 4A, DL blocked the EPS production in matured *C. albicans* biofilms, in the concentration-dependent way. 16, 32 and 64 µg/mL of DL can decrease EPS by roughly 31%, 44% and 60%. EPS contribute to the resistance of *C. albicans* biofilms to antifungals and Fig. 4A may indicate that DL can sensitize *C. albicans* biofilms to antifungal drugs. As shown in Fig. 4B, DL could suppress the phospholipase production in the strain SC5314. In spite of the narrow increase in Pz, the differences between treatments and controls were significant. As phospholipase contributes to *C. albicans* infectivity, the results of Fig. 4B may also suggest that DL could suppress *C. albicans* infectivity in animals.

### DL causes the cell membrane damages in ***C. albicans***

**Fig. 5:**
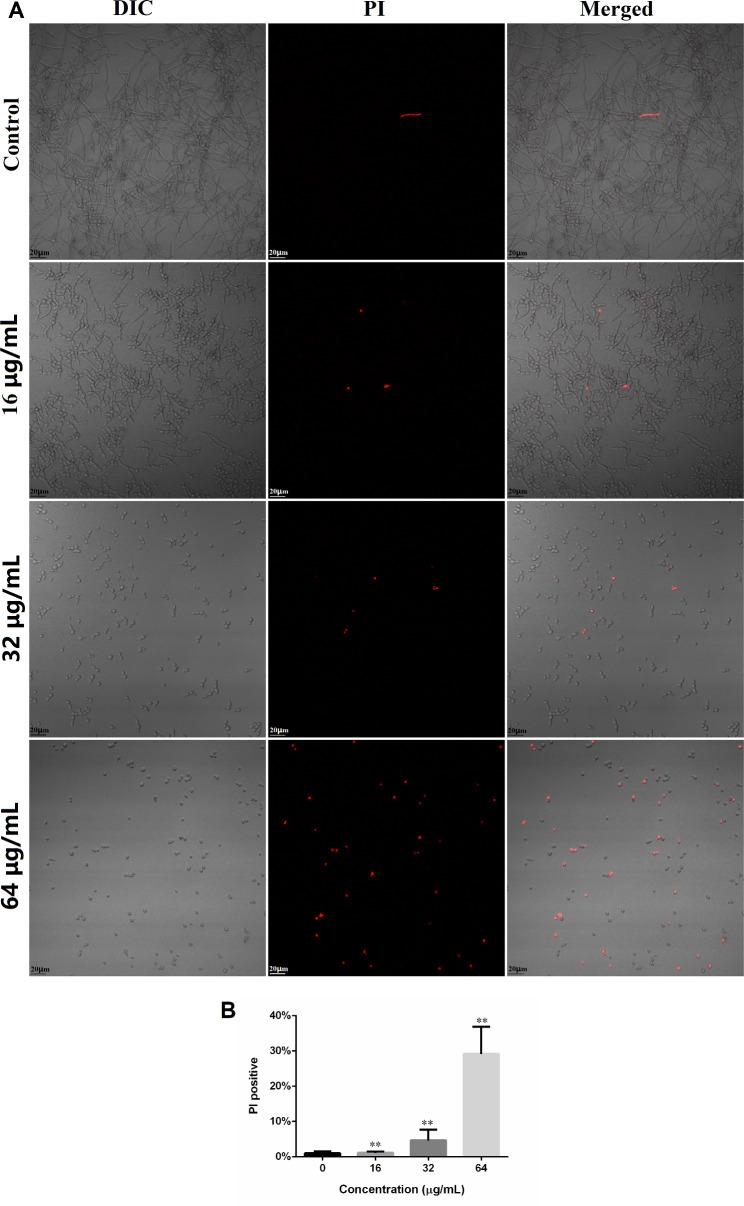
DL induced damages to the cell membrane of *C. albicans*. (**A**) *C. albicans* cells exposed to 0, 16, 32 and 64 µg/mL DL for 4 h, were stained with PI (final concentration: 10 µM) for 20 min in dark and photographed by CLSM. (**B**) The percentages of PI positive cells in each group. **, *p* < 0.01

Cell membrane is critical for most organisms in that they can prevent harmful materials into cytoplasm while they can import nutrients actively. Figure 5 A showed that DL can increase the number of *Candida* cells with damaged cell membrane which allows PI (a cell membrane-impermeable dye) to access into cells and emit red fluorescence. However, the differences between control and 16 µg/mL DL were insignificant, but the treatment with 32 and 64 µg/mL DL significantly increased the PI positive cells (Fig. 5B).

### DL caused excessive ROS production

**Fig. 6:**
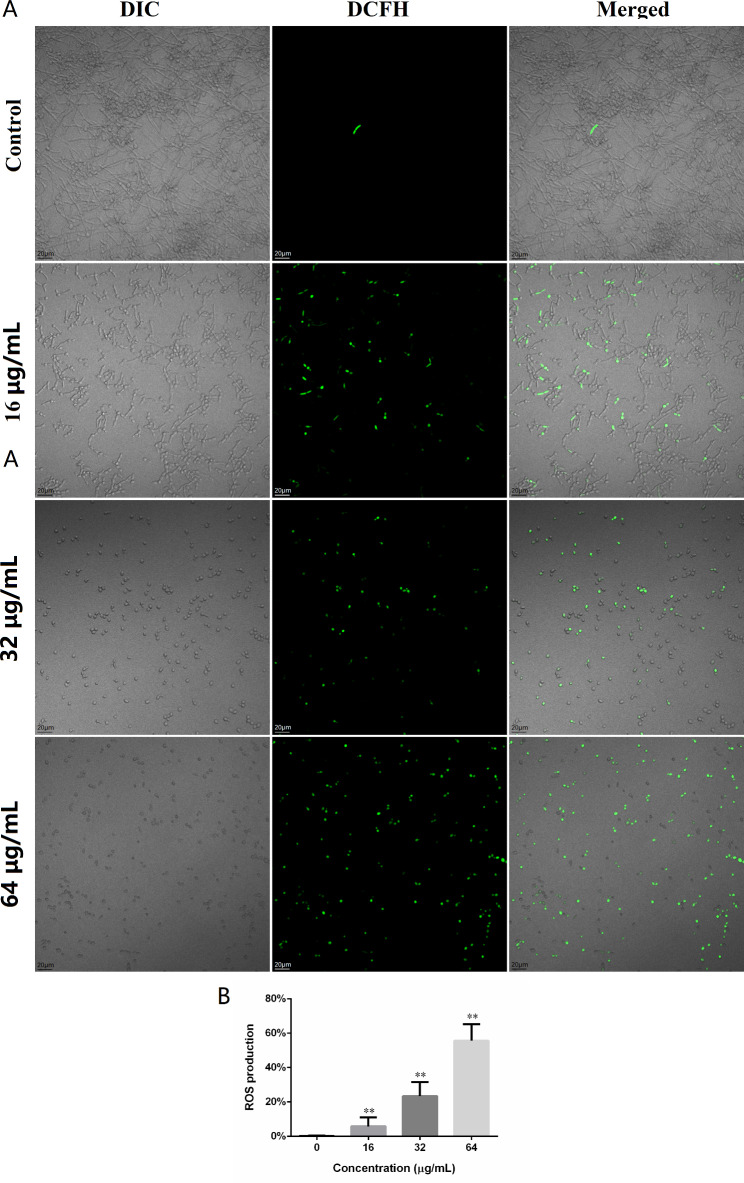
DL increased the ROS production in *C. albicans* SC5314. (**A**) *C. albicans* cells exposed to 0, 16, 32 and 64 ug/mL DL for 4 h, were stained with DCFH-DA (final concentration: 10 µM) for 20 min in dark and were subjected to CLSM for observation. Green fluorescence indicated the increased intracellular ROS. (**B**) The statistics of ROS production in each group. **, *p* < 0.01

The intracellular ROS production were evaluated by DCFH-DA staining after an incubation with DL for 4 h. As shown in Fig. 6A, DL treatment triggered concentration-dependent ROS generation in *Candida* cells. The ROS production in groups treated by 16–64 µg/mL of DL was significantly higher than that in negative control (Fig. 6B). These excessive ROS may cause oxidative damages to cell membrane and bio-macromolecules of *C. albicans* cells.

### NAC rescue experiment

To confirm that ROS contribute to the anti-*Candida* activity of DL, 150 µg/mL NAC was supplemented in the assays of MIC, morphological transition, biofilm formation. NAC doubled the MIC of DL against *C. albicans* SC5314 (Table 2), indicating the involvement of ROS in the antifungal activity of DL.

**Table 2:** Effects of antioxidant NAC on the growth of *C. albicans* SC5314 in the presence of DL

DL concentration (µg/mL)	Without 150 µg/mL NAC	With 150 µg/mL NAC
256	+++ +++ +++	+++ +++ +++
128	+++ +++ +++	+++ +++ +++
64	+++ +++ +++	--- --- ---
32	--- --- ---	--- --- ---

+, no fungal growth; -, fungal growth. The number of + or – represents the repeat times

**Fig. 7:**
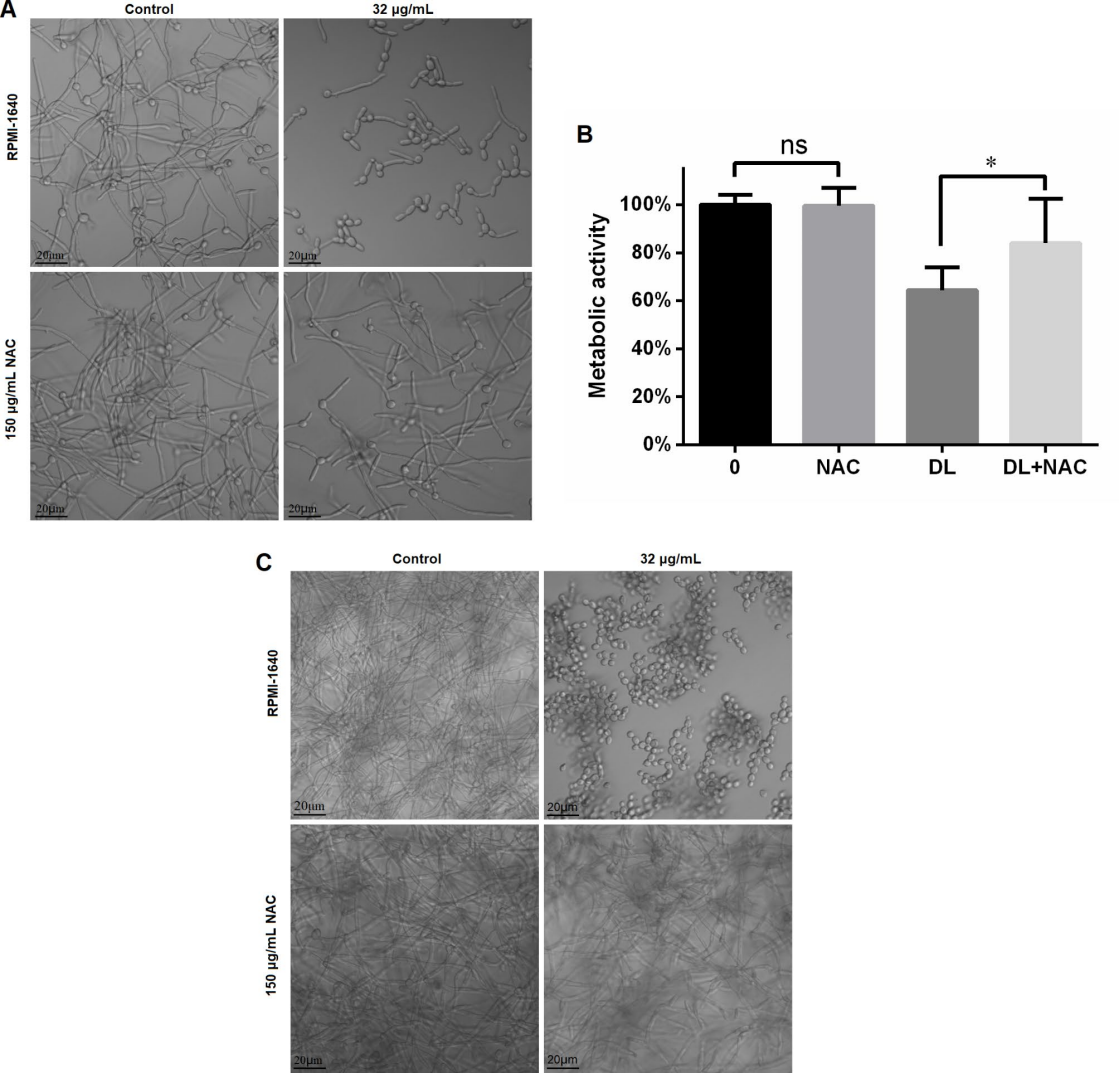
The presence of NAC could partly rescue the inhibition of DL on hyphal growth and biofilm formation of *C. albicans* SC5314. (**A**) 150 µg/mL NAC could rescue the inhibitory effects of DL on *C. albicans* hyphal formation. (**B**) 150 µg/mL NAC could rescue the inhibitory effects of DL on the metabolic activity of (**C**) *albicans* SC5314. DL was added, with or without 150 µg/mL NAC, to *C. albicans* suspensions that were used to form biofilms for 24 h, followed by XTT assay. *, *p* < 0.05. C. The *C. albicans* biofilms formed with or without DL, in the presence or absence of 150 µg/mL NAC were photographed

We further investigated whether the effects of DL could be saved by NAC. As expected, the presence of NAC could rescue the hyphal inhibition of DL (Fig. 7A). The supplementation of NAC could also attenuate the suppression of DL on biofilm formation, as shown in Fig. 7B C.

## Discussion

*Candida* infections have increased in the past decades. Particularly, in intensive care units, *C. albicans* is associated with significant morbidity of skin and mucosal infections and with formidable candidemia that is often nosocomial and highly mortal (Qian et al. 2020). Currently, there are only several antifungal drugs available while the drug resistance is increasing (Liu et al. 2017). The biofilms of this fungus on medical materials (central vein catheters etc.) are resistant to antifungals and cause persistent infections (Ibrahim et al. 2019). Therefore, it is pressing to seek novel antifungal agents. Especially, agents active against biofilms are needed. Here, we reported that DL showed significant antifungal activity and suppressed *C. albicans* adhesion, morphological transition and biofilms.

Although DL failed to demonstrate antifungal activity against *C. albicans* in one report in 2019 (Ibrahim et al. 2019), we and other groups did confirm that DL has anti-*Candida* activity in multiple *C. albicans* strains and isolates (Neves et al. 1999; Shin and Kim, 2016). The discrepancies in the results may stem from different strains and methods used by different groups (Shin and Kim, 2016; Ibrahim et al. 2019). Our results also expand the antifungal spectrum to include *C. krusei*, which is an intrinsically fluconazole-resistant *Candida* pathogen (Jamiu et al. 2021).

Morphological transition to hyphae, the main virulence factors of *C. albicans*, enables this fungus to invade host tissues, cause damages and survive in phagocytes (Bain et al. 2021; Siscar-Lewin et al. 2022). Hyphae can express adhesins and secreted aspartic proteinases that may facilitate the adhesion and invasion of *C. albicans* (Siscar-Lewin et al. 2022). *C. albicans* hyphae also secrete the exotoxin candidalysin to cause damages to epithelial or immune cells, triggering pro-inflammatory IL-1β release (Naglik et al. 2019). DL blocked the hyphal growth of *C. albicans* and its adhesion, suggesting that DL has the potential to prevent the adhesion and invasion of *C. albicans* in hosts. *C. albicans* overgrowth, hyphae formation and candidalysin secretion, has been confirmed to play important roles in alcoholic liver disease and ulcerative colitis, one type of inflammatory bowel disease, through enhancing the production of IL-1β (by activating dectin-1/NF-κB pathway and NLRP3 inflammasomes) which aggravates inflammation (Yang et al. 2017; Chu et al. 2020; Li et al. 2022). Candidalysin may also direct damage the epithelial cells (Moyes et al. 2016; Allert et al. 2018). In addition, high burden of intestinal *Candida* before fecal microbiota transplantation (FMT) may predict good outcomes of FMT in patients with ulcerative colitis (Leonardi et al. 2020), suggesting that in ulcerative colitis patients, inhibition of *Candida* may be beneficial. DL and the DL-containing food (Jangkanghwan), herb (*Vladimiriae Radix* and *Aucklandia lappa* Decne) or formula (Xianglian pill and KM1608), have demonstrated efficacy in dextran sulfate sodium (DSS)-induced colitis through multiple mechanisms including suppressing IKKα/β-NF-κB and activating Nrf2 pathway (Lee et al. 2020; Long et al. 2020; Liu et al. 2021a; Yu et al. 2021; Chen et al. 2022; Yuan et al. 2022). In DSS-induced colitis, DL can reduce the ulcerative colitis-related colorectal inflammation, including significant reduce in IL-1β (Zhou et al. 2020). However, these studies were performed in mice without introducing *C. albicans* into gut (Lee et al. 2020; Long et al. 2020; Zhou et al. 2020; Liu et al. 2021a; Yu et al. 2021; Chen et al. 2022; Yuan et al. 2022). Because *C. albicans* is one member of human gut microbiota but not one of mice (Liu et al. 2021b), it is possible that the suppression of DL on *C. albicans* growth, hyphal formation and candidalysin secretion (thus inhibiting IL-1β release) may contributed to its efficacy in ulcerative colitis in human or humanized mice that include *C. albicans* as a gut commensal fungus (Li et al. 2022). Consistent with our proposal, paeonol was reported to alleviate *C. albicans*-associated colitis induced by DSS in mice through suppressing dectin-1/NF-κB signaling in combination with TLR2 and TLR4 (Ge et al. 2021), while the antifungal drug fluconazole treatment could rescue the ulcerative colitis symptoms in mice (Leonardi et al. 2018). However, the potential roles of DL proposed here needs to be further identified through well-designed experiments.

*C. albicans* biofilm-related infections on medical materials are refractory while the intertwined hyphae within biofilms strengthen the biofilm structures (Blankenship and Mitchell, 2006; Liu et al. 2017). The fact that DL can inhibit the growth and the yeast to hyphal transition inspired us to test its effects on *C. albicans* biofilms. As showed by XTT and CLSM results, DL can suppress both the biofilm formation and development, which may indicate the potential use of DL in the *C. albicans* biofilm-related infections.

EPS may hinder the fungal cells within biofilms from being attacked by drugs, thus reducing the efficacy of antifungal drugs (Yang et al. 2018c). Our results that DL can suppress *C. albicans* biofilm EPS generation suggested that DL may help other antifungal drugs to reach their intracellular targets within biofilms, producing synergistic or additive effects when combined with other antifungal drugs, which needs further experimental validation. Consistent with this proposal, the combination of AmB and the EPS-inhibiting alantolactone produce an additive interaction (Yang et al. 2022b). Data from animal models suggested that the extracellular phospholipase produced by *C. albicans* plays important roles in its pathogenicity through breaking down the phospholipids in cells, while mutants with defects in phospholipase showed attenuated infectivity (Singh et al. 2018; El-Baz et al. 2021). The effect of DL on extracellular phospholipase indicated that DL may reduce the infectivity of *C. albicans*. In a word, DL can inhibit the growth and virulence factors of *C. albicans*.

Furthermore, we explored the preliminary antifungal mechanisms and found that DL caused damages to fungal cell membrane, leading to higher cell membrane permeability, which can also be seen in other antifungal agents (Yang et al. 2018a, 2022b; Gowri et al. 2020). Physiological level of ROS can act as signal molecules and be degraded by superoxidases and catalase (Wang et al. 2021). Once this balance is disrupted, the overproduction of intracellular ROS occurs, causing damages to membranes and biomacromolecules, which may lead to metabolic disturbances and cell death (Gong et al. 2019). Since DL has showed ROS-inducing activity in leukemia cells and renal cells (Cai et al. 2017; Singireesu et al. 2018), it is proposed that DL may cause excessive ROS production in *C. albicans* cells. As supposed, DL significantly increased the intracellular ROS production while the supplementation of antioxidant NAC could rescue the planktonic growth and biofilm formation, as well as the hyphal formation, that could be inhibited by DL, which further confirmed the involvement of ROS. However, whether the DL-caused ROS result in apoptosis of *C. albicans*, like antifungal agents naringin and chelerythrine (Gong et al. 2019; Kim and Lee, 2021), remains to be explored in the future.

Although DL showed obvious antifungal activity against multiple *C. albicans* strains and inhibited *C. albicans* virulence factors, it should be noted that the cytotoxicity in mammalian cells was also non-negligible since theIC_50_ against renal cells was at the level of µM (Singireesu et al. 2018), which may be related to its good antitumor activities (Jiang et al. 2015; Zhang et al. 2020). While the existence of DL-containing food and herb, may indicate its safety in practices, confronting the in vitro cytotoxicity mentioned above (Lee et al. 2020; Long et al. 2020; Liu et al. 2021a). Other limitations of this study include the lack of deep mechanism and in vivo therapeutic efficacy of DL. Therefore, much more work (such as deep mechanism exploration, structural optimization and animal tests) are needed to be done, before this compound or its analogs can come to use in clinic.

In conclusion, our study showed that DL inhibits not only the planktonic growth of *C. albicans* and other *Candida* species, but also the morphological transition, adhesion and biofilms of *C. albicans*. The anti-*C. albicans* activity of DL may involve damages to cell membrane and overproduction of intracellular ROS. DL might be exploited to develop antifungal therapies against infections caused by *Candida* species.

## Data Availability

Available from the corresponding author.
